# Management of odontogenic sinusitis related to dental implants and maxillary sinus grafting: a retrospective single-center study

**DOI:** 10.3389/fsurg.2026.1862910

**Published:** 2026-06-26

**Authors:** Lorenzo Sabatino, Michele Antonio Lopez, Marco Mattaroccia, Matteo Valentini, Francesco Iafrati, Luigi De Benedetto, Antonio Moffa, Manuele Casale

**Affiliations:** 1 Unit of Otolaryngology, Fondazione Policlinico Universitario Campus Bio-Medico, Rome, Italy; 2 Specialised Odontostomatology Department of Head and Neck and Sensory Organs, Division of Oral Surgery and Implantology, Fondazione Policlinico Universitario A. Gemelli IRCCS-Università Cattolica del Sacro Cuore, Rome, Italy; 3 Unit of Otolaryngology, Department of Medicine and Surgery, Università Campus Bio-Medico di Roma, Rome, Italy

**Keywords:** complications, dental implants, FESS, maxillary sinus grafting, odontogenic sinusitis, oroantral communication, peri-implantitis

## Abstract

**Background:**

The increasing use of dental implants is changing the epidemiology of Odontogenic Sinusitis (ODS). Aside from endodontic causes, we frequently encounter problems caused by implant placement and bone augmentation techniques, which present a distinct pathophysiological profile compared to classic ODS. This study analyzed the clinical features of pre-implant (Group I) and implant-related ODS (Group II) to establish surgical management strategies according to the Felisati classification.

**Methods:**

This retrospective single-center study analyzed 45 patients treated between December 2020 and December 2025. Patients were classified according to the Felisati classification into Group I (preimplantological treatment complications) and Group II (implantological treatment complications). We evaluated clinical variables -etiology, osteomeatal complex (OMC) obstruction, foreign body presence, and oroantral communications (OAC)—and surgical strategies adopted: Endoscopic Sinus Surgery (FESS), exclusive oral approach, or a combined simultaneous technique.

**Results:**

Implant-related cases (Group II; *n* = 37) dominated the cohort (82.2%), with 8 preimplantological complications (Group I). Females accounted for 66.7%. In Group II, chronic sinusitis affected 70.3% of patients, with high incidence of foreign bodies and OACs (64.9% each). To manage this complex scenario, we favored a combined simultaneous approach in the majority of Group II cases (59.5%); we reserved the exclusive oral approach for 27.0% of patients and limited exclusive FESS to 13.5%, primarily for implant preservation. Similarly, in Group I, where chronic sinusitis affected 75% of patients, the combined approach was performed in half the cases, while FESS alone was utilized in 37.5% to retrieve dispersed graft material. At the 3-month follow-up, primary complete resolution was observed in 44/45 patients (97.8%). Only one patient (Group II) experienced a recurrence of the oro-antral communication, which was successfully closed after a secondary surgical intervention, ultimately leading to a 100% final success rate.

**Conclusions:**

Implant-related and pre-implant ODS are distinct clinical entities often complicated by OMC obstruction and foreign body reactions. Although surgical planning requires case-by-case customization, in our experience, the combined simultaneous approach represented an effective primary strategy for complex cases, while exclusive approaches were reserved for selected patients. Complete short-term clinical and endoscopic resolution was observed in the cohort at the 3-month follow-up.

## 1 Introduction

Maxillary sinusitis of odontogenic origin (ODS) represents a distinct infectious entity of the paranasal sinuses arising from dental pathologies or sequelae of dental procedures. While historically estimated to account for a minor fraction (10%–12%) of maxillary sinusitis cases (1, 2), contemporary data suggest a significant epidemiological shift, with ODS now potentially responsible for 30%–40% of chronic cases, which typically present unilaterally (3, 4). This condition is pathophysiologically distinct from rhinogenic sinusitis, primarily due to the mechanical breach of the Schneiderian membrane and a unique microbiological profile characterized by a polymicrobial flora with a marked prevalence of anaerobes (2, 4). The frequent failure to recognize the underlying dental etiology often results in the inefficacy of standard medical treatments and the subsequent chronicization of the disease (3).

To establish a standardized framework for diagnosis and management, Felisati et al. (2013) introduced an outcome-oriented clinical classification that categorizes sinonasal complications based on their specific pathogenesis (1). This system delineates three main categories: **Group I** – preimplantological treatment complications – encompasses complications arising from pre-implantological procedures, such as sinus floor elevation; **Group II** – implantological treatment complications - involves complications associated with implant placement, including displacement or peri-implantitis; and **Group III - “**Classic” dental treatment complications **-** refers to complications stemming from “classic” dental pathologies, such as endodontic infections or oroantral communications (OAC) (1). Such a classification is indispensable for coordinating a multidisciplinary therapeutic strategy involving both otolaryngologists and oral surgeons (1, 3).

The surge in dental implant rehabilitation has inevitably led to a rising incidence of implant-related odontogenic sinusitis (falling into Felisati's Group II). This variant of ODS may be precipitated by the implant penetrating the sinus cavity—acting as a foreign body or a conduit for infection—or by peri-implantitis spreading apically to cause osteitis and subsequent sinus infection (5, 6). Notably, recent literature indicates a significantly higher prevalence of peri-implantitis in sites affected by implant-related sinusitis compared to healthy controls (5). Additionally, the accidental migration or displacement of dental implants into the sinus remains a critical complication often necessitating surgical retrieval to resolve the infectious process (4, 6).

Alongside implant therapy, the management of atrophic maxillae frequently requires bone augmentation techniques such as sinus lifts. These procedures can give rise to sinusitis of pre-implant etiology (“preimplantological treatment complications”). Although these procedures generally boast low complication rates, issues such as graft infection, membrane perforation, or graft displacement can trigger refractory chronic sinusitis (7). In such scenarios, the pathophysiology is driven by the superinfection of the grafting material, which acts as a bacterial reservoir within the sinus, disrupting homeostasis and demanding complex management (1, 7).

Therapeutically, ODS - whether of implant or pre-implant origin - often dictates a combined medical and surgical protocol, as antibiotic therapy alone is ususally not sufficient to eradicate the polymicrobial infection in the presence of foreign bodies or infected bone (5, 7).

The objective of this retrospective study is to describe our single-center surgical experience and analyze the specific clinical and anatomical factors associated with the choice of surgical approach in managing ODS related to dental implants and sinus floor elevation.

## 2 Materials and methods

This retrospective single-center study evaluated the clinical data of patients who underwent surgical treatment for odontogenic sinusitis at the Unit of Integrated Therapies in Otolaryngology (Campus Bio-Medico University Hospital, Rome, Italy) between December 2020 and December 2025. A retrospective study was conducted analyzing the collected clinical (etiology, type of treatment, complications) and demographic (age, sex) data.

### 2.1 Inclusion criteria

The inclusion criteria applied for the review of clinical cases were modeled after recent protocols for odontogenic sinusitis management (3):

1. Clinical diagnosis of odontogenic sinusitis, supported by radiological and/or endoscopic findings (3, 6), and classified according to Felisati et al. (1) into Group I (complications following maxillary sinus grafting prior to or in association with oral implant placement) or Group II (complications following the use of oral implants in the upper jaw).
2. Resistance to medical treatment, defined as the persistence of symptoms and endoscopic signs of infection after at least two 10 to 14 day cycles of broad-spectrum oral antibiotics (e.g., amoxicillin/clavulanic acid or fluoroquinolones) (3).
3. Multidisciplinary specialist agreement (ENT and maxillofacial surgeon/dentist) on the odontogenic focus (1, 3).
4. Presence of Computed Tomography (CT) of the maxillofacial complex performed prior to surgical intervention (3, 4).

### 2.2 Exclusion criteria

Patients with a history of pre-existing chronic rhinosinusitis, with or without nasal polyps (CRSwNP or CRSsNP), unrelated to the dental condition, and patients belonging to Group III (classic non-implant dental pathologies) (1, 3).

### 2.3 Study population and patient flow

During the study period, a total of 160 patients presenting with odontogenic sinusitis were initially evaluated at our institution. Following the strict application of our inclusion and exclusion criteria, 115 patients were excluded as they belonged to Felisati Group III, yielding a final highly selected cohort of 45 patients eligible for the present analysis.

### 2.4 Diagnostic workup

To reach the clinical diagnosis, signs and symptoms typically associated with this pathology (anterior and/or posterior purulent rhinorrhea, nasal obstruction usually unilateral, maxillary pain, cacosmia) arising subsequent to implantology or pre-implant surgery (e.g., sinus lift) were considered (3, 7). During the first ENT visit, nasal endoscopy was performed using a flexible or rigid endoscope to explore the nasal fossae and osteomeatal complexes (3). An accurate examination of the oral cavity was carried out to identify gingival lesions, oroantral communications (OAC), or implant exposures. The diagnosis was confirmed by analysis of the CT scan (without contrast medium), which is fundamental for evaluating the extent of the sinus pathology, the presence of foreign bodies (e.g., displaced implants, dispersed graft material), and potential bone erosion (3, 8).

In cases presenting with displaced macroscopic foreign bodies or oroantral communications (OAC), medical therapy was primarily administered as a preparatory phase to reduce acute mucosal inflammation prior to necessary surgical intervention, rather than as a definitive curative attempt.

### 2.5 Bias assessment

To minimize measurement and observer bias, all diagnostic modalities were subjected to a rigorous multidisciplinary review. Specifically, preoperative computed tomography (CT) scans were independently assessed by at least three specialists (a radiologist, an otolaryngologist, and an oral surgeon), while preoperative and postoperative endoscopic findings were evaluated jointly by at least two specialists (an otolaryngologist and an oral surgeon).

### 2.6 Surgical procedure

Surgical treatment was planned based on the Felisati classification (1) and the specific clinical presentation. Surgical options included an exclusive nasal approach (FESS), an exclusive oral approach, or a combined (simultaneous) approach (1, 3).

FESS (Functional Endoscopic Sinus Surgery) was performed under general anesthesia using 0°, 30°, 45°, and 70° rigid endoscopes (3). The procedure was preceded by nasal decongestion using cottonoids soaked in local vasoconstrictor. The standard intervention involved uncinectomy followed by a wide middle maxillary antrostomy to ensure ventilation and drainage of the sinus and to allow the removal of any foreign bodies (e.g., graft fragments or migrated implants) or mycetomas accessible via the endoscopic route (1, 3, 7). Any obstructive anatomical variations (e.g., septal deviation, concha bullosa) were corrected contextually (3).

An oral or combined approach was adopted when it was necessary to remove implants or graft materials that were inaccessible via the nasal route, or to treat specific complications such as oroantral communication (OAC) (1, 3, 6). In the presence of OAC, plastic closure of the communication was performed (e.g., vestibular sliding flap or use of the buccal fat pad) (3). In our sample, the choice of approach was guided by the need to remove the pathogenic *noxa* (infected or displaced implant, superinfected graft material) and restore sinus homeostasis (1, 8). The specific operational clinical and radiological criteria utilized for selecting each surgical strategy, as well as the explicit indications for dental implant preservation or removal, are systematically detailed in Table 3.

### 2.7 Post-operative care

Patients were discharged on the 1st or 2nd post-operative day, depending on the extent of the intervention. The post-operative protocol included antibiotic therapy (generally amoxicillin with clavulanic acid or fluoroquinolones in case of allergy or specific indication) for 7–10 days and nasal washes with saline solution (3, 4).

### 2.8 Outcome definition

Treatment success was defined as the complete resolution of symptoms and the absence of endoscopic signs of sinusitis at post-operative controls performed at 30 and 90 days after the intervention (3).

### 2.9 Statistical analysis

A descriptive analysis of demographic and clinical variables was performed. Categorical variables were reported as frequencies and percentages, while continuous variables were expressed as mean and range. To evaluate the clinical efficacy of the proposed decision-making algorithm, the overall success rate was computed alongside its 95% confidence interval (CI) using the Clopper-Pearson exact method. Statistical analyses were performed using STATA 18 Software (StataCorp LLC, Lakeway Drive College Station, Texas, USA)

### 2.10 Ethics statement

The study was conducted in accordance with the Declaration of Helsinki. Ethical review and approval were waived for this study due to its retrospective observational nature, based exclusively on the analysis of pre-existing routine clinical data.

## 3 Results

A total of 160 patients with a diagnosis of odontogenic sinusitis were screened at our Institution during the study period. Among these, 115 patients were excluded as they belonged to Felisati Group III. The final study cohort consisted of 45 consecutive patients requiring surgical intervention for Group I (*n* = 8) or Group II (*n* = 37) complications. Of these, 15 were men (33.3%) and 30 were women (66.7%). All patients presented with a clinical history of prior implant placement or pre-implant augmentation procedures, and the odontogenic origin of the sinusitis was confirmed by clinical records, endoscopic findings, and radiological evidence (CT scan).

The most frequently reported symptoms included unilateral nasal obstruction, purulent rhinorrhea, facial pain/pressure, and cacosmia. All patients had failed prior medical management, which included at least two 10-to 14-day courses of broad-spectrum oral antibiotics (e.g., amoxicillin/clavulanic acid or fluoroquinolones), topical therapy consisting of 0.9% saline nasal washes at least three times daily and topical corticosteroid two times daily for 20 days. Pre-operative CT scans revealed unilateral sinus opacification in all cases. An obstruction of the osteomeatal complex (OMC) was reported in 31 patients (68.9%).

Regarding the specific etiology classified according to Felisati et al.:

- Implantological treatment complications (Group II) were identified in 37 patients (82.2%).
- Preimplantological treatment complications (Group I), such as infected sinus grafts, were identified in 8 patients (17.8%).

### 3.1 Preimplantological treatment complications (group I)

The cohort of patients with pre-implant odontogenic sinusitis (Felisati Group I) consisted of 8 subjects.

**Figure 1:**
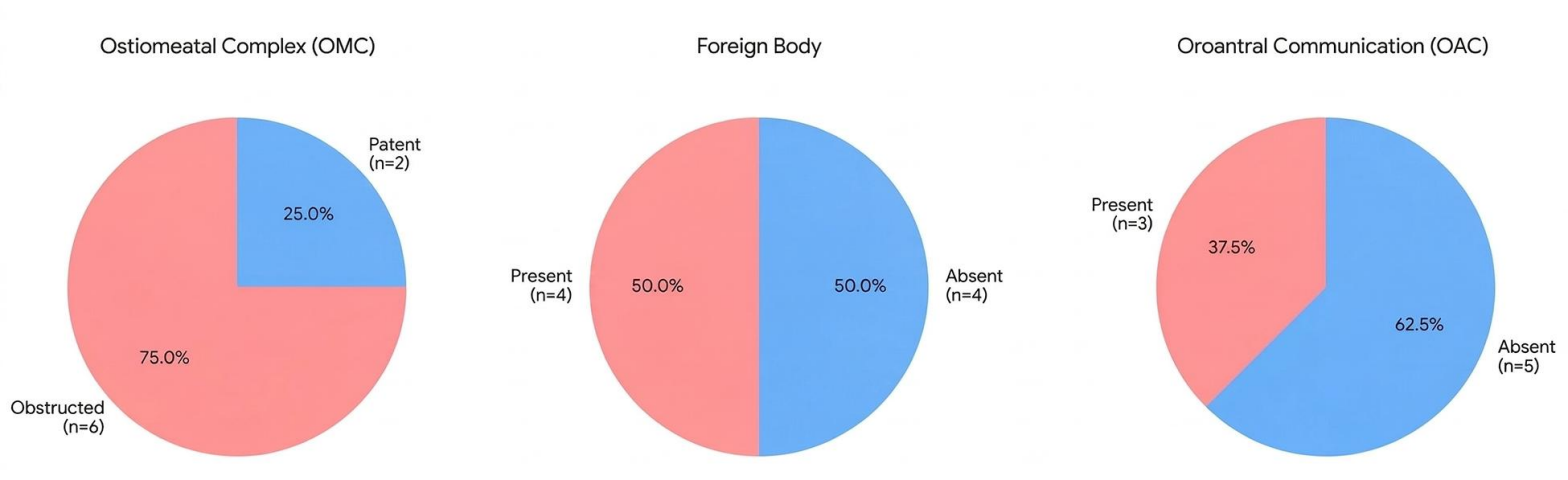
Anatomical findings in group I (preimplantological treatment complications).

- Clinical Phenotype: Chronic sinusitis was the predominant presentation, observed in 6 patients (75%), while acute sinusitis was found in 2 patients (25%).
- Anatomical Findings: Obstruction of the osteomeatal complex (OMC) was documented in 6 patients (75%), whereas the OMC was patent in the remaining 2 (25%). A foreign body within the maxillary sinus was detected in 4 cases (50%). An oroantral communication (OAC) was present in 3 patients (37.5%) (Figure 1) (Table 1).
- Surgical Management: The most frequent treatment was the combined approach, performed in 4 patients (50%). An exclusive nasal approach was utilized in 3 cases (37.5%), and an exclusive oral approach was selected for 1 patient (12.5%) (Table 2).

Regarding the pre-implantological complications in Group I (*n* = 8), precise documentation detailing the exact commercial or chemical composition of the graft material was not systematically available due to the retrospective design of our study.

**Table 1:** Baseline demographics and clinical characteristics of the cohort.

Characteristic	Group I (*n* = 8)	Group II (*n* = 37)	Total Cohort(*N* = 45)
Age (years, mean ± SD)	57.5 ± 13.2	60.3 ± 14.7	59.8 ± 14.4
Gender, n (%)			
- Female	5 (62.5%)	25 (67.6%)	30 (66.7%)
- Male	3 (37.5%)	12 (32.4%)	15 (33.3%)
Side of Involvement, n (%)			
- Right	3 (37.5%)	16 (43.2%)	19 (42.2%)
- Left	5 (62.5%)	21 (56.8%)	26 (57.8%)
Clinical Presentation, n (%)			
- Chronic Sinusitis	6 (75.0%)	26 (70.3%)	32 (71.1%)
- Acute/Recurrent Sinusitis	2 (25.0%)	8 (21.6%)	10 (22.2%)
- Foreign Body without Sinusitis	0 (0.0%)	3 (8.1%)	3 (6.7%)
Radiological & Endoscopic Findings, n (%)			
- Osteomeatal Complex (OMC) Obstruction	6 (75.0%)	25 (67.6%)	31 (68.9%)
- Oroantral Communication (OAC)	3 (37.5%)	24 (64.9%)	27 (60.0%)
Characterization of Septic Foreign Body, n (%)			
- Displaced Maxillary Graft Biomaterial	4 (50.0%)	—	4 (8.9%)
- Septic Dental Implant	—	24 (64.9%)	24 (53.3%)

**Table 2:** Surgical approaches and clinical outcomes at 3-month follow-Up.

Outcome Measure	Group I (*n* = 8)	Group II (*n* = 37)	Total Cohort(*N* = 45)
Surgical Strategy adopted, n (%)			
- Combined Simultaneous approach (FESS + Oral)	4 (50.0%)	22 (59.5%)	26 (57.8%)
- Exclusive Oral Approach	1 (12.5%)	10 (27.0%)	11 (24.4%)
- Exclusive Endoscopic Approach (FESS)	3 (37.5%)	5 (13.5%)	8 (17.8%)
Follow-up Duration	3 Months	3 Months	3 Months
Therapeutic Efficacy, n (%)			
- Primary Complete Resolution (1st surgery)	8 (100.0%)	36 (97.3%)	44 (97.8%)
- Persistent OAC/Postoperative Recurrence	0 (0.0%)	1 (2.7%)	1 (2.2%)
- Success after Secondary Revision Surgery	—	1 (100.0%)	1 (100.0%)
Final Overall Clinical Success Rate, n (%)	8 (100.0%)	37 (100.0%)	45 (100.0%)

### 3.2 Implantological treatment complications (group II)

The cohort of patients with implant-related odontogenic sinusitis (Felisati Group II) comprised a total of 37 subjects.

**Figure 2:**
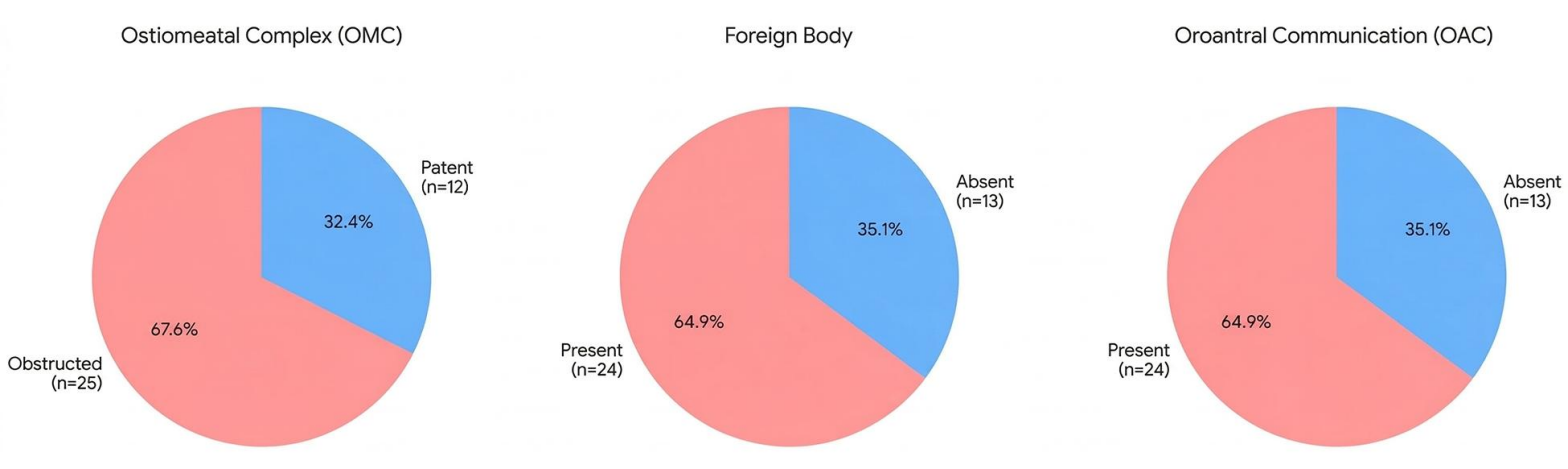
Anatomical findings in group II (implantological treatment complications).

- Clinical Phenotype: The majority of patients presented with chronic sinusitis (*n* = 26; 70.3%). Acute sinusitis was observed in 8 patients (21.6%), while 3 patients (8.1%) presented with a foreign body reaction without frank sinusitis.
- Anatomical Findings: An intra-sinus foreign body was identified in 24 patients (64.9%). Similarly, obstruction of the osteomeatal complex (OMC) was reported in 25 patients (67.6%). An oroantral communication (OAC) was present in 24 cases (64.9%) (Figure 2) (Table 1).
- Surgical Management: Surgical treatment primarily involved a combined approach, utilized in 22 patients (59.5%). An exclusive oral approach was performed in 10 patients (27.0%), while an exclusive nasal approach was adopted in 5 patients (13.5%) (Table 2).

Regarding the fate of the dental implants in Group II (*n* = 37), a total of 24 implants (64.9%) were surgically removed, while the remaining 13 implants (35.1%) were successfully preserved or involved the management of previously explanted sites.

Intraoperative microbiological cultures were not routinely or systematically performed for the patients in this cohort. Post-operatively, no major complications were recorded. All patients received standard antibiotic prophylaxis. Clinical and endoscopic follow-up at 3 months confirmed complete resolution of symptoms, endoscopic patency, and mucosal healing in 44 out of 45 patients yielding a primary success rate of 97.8% (95% CI: 88.2% – 99.9%). Only one patient belonging to Group II (2.2%), who initially presented with an oro-antral communication (OAC) managed via a combined approach, experienced a reopening of the fistula at the 3-month evaluation. This recurrence required a secondary surgical intervention for OAC closure, after which complete and stable resolution was achieved, resulting in a final overall success rate of 100% (95% CI: 92.1% – 100.0%).

## 4 Discussion

The management of odontogenic sinusitis (ODS) has been significantly structured by the classification proposed by Felisati et al. (1), which provides a reliable framework for categorizing these infections. While this classification is widely accepted, clinical reports focusing specifically on the detailed phenotypic characteristics of Group I (graft-related) and Group II (implant-related) cases can offer further insights into surgical decision-making. Given the increasing frequency of these specific complications in modern dental practice, we reviewed our institutional experience with 45 patients belonging to these two categories. Our objective was to describe the specific clinical presentations—such as the state of the osteomeatal complex (OMC), the presence of an oro-antral communication (OAC), or the migration of foreign materials—and to observe how these individual phenotypic features influenced the choice between an exclusive oral, exclusive nasal, or combined simultaneous surgical approach. Through the analysis of these distinct phenotypic profiles, we sought to define a more tailored surgical management, aiming to identify the most appropriate treatment strategy for each individual case.

This retrospective analysis strictly included patients with odontogenic sinusitis related to Group I and Group II. Therefore, the proportions of etiologies in our cohort—predominantly Group II (implant-related, 82.2%) and secondarily Group I (pre-implant-related, 17.8%)—reflect our specific patient selection criteria rather than a general epidemiological picture. Nonetheless, this targeted cohort enables a more thorough insight into this particular clinical entity, which differs substantially from classic odontogenic sinusitis (4, 5). Consistent with existing literature on implant-seeking populations, demographic analysis of our sample showed a female preponderance (66.7%).

### 4.1 Preimplantological treatment complications (group I)

#### 4.1.1 Graft properties and the prevention of pathophysiological complications

While precise documentation detailing the exact commercial or chemical composition of the graft material was not systematically available in our cohort, the physical consistency and granulometry of these materials theoretically dictate the severity of complications upon sinus migration. Large-granule formulations, if displaced, often fail to clear naturally and become mechanically entrapped in the osteomeatal complex (OMC), causing a “dam effect” and complete obstruction of sinus ventilation (9, 10). Understanding these specific material risks is vital for prevention during case selection and explains the absolute need for endoscopic clearance (FESS) when macroscopic displacement occurs.

To stabilize the graft adjacent to the residual bone walls and potentially reduce the risk of material displacement, several techniques have been described, including the Tulasne technique (which utilizes cortical bone to create a new maxillary sinus floor) or, as previously described by our group (11), the application of a thick resorbable collagen membrane combined with a xenogeneic dual-phase bone substitute to seal the antrostomy (the “Sinus Pack” technique). This methodology provides an additional protective layer that helps prevent odontogenic sinusitis during maxillary grafting procedures. It is particularly valuable as it enhances protection and mitigates the risk of complications, effectively managing both intraoperative Schneiderian membrane perforations and potential delayed post-operative membrane failures. While these preventative techniques aim to avoid the material displacement observed in our Group I cohort, once macroscopic displacement and superinfection occur, surgical retrieval becomes mandatory.

#### 4.1.2 Osseointegration and foreign body reactions

Furthermore, the absence of osseointegration converts the graft into a non-vital foreign body. Autologous bone will resorb physiologically, but synthetic or xenogenic particles (especially non-resorbable products such as some bovine bone derivatives or hydroxyapatite) remain *in situ* indefinitely. In the event of infection, these particles serve as a platform for bacterial biofilm formation that maintains the chronic inflammatory state, subsequently requiring surgical excision using FESS to re-establish sinus homeostasis (10, 12).

### 4.2 Implantological treatment complications (group II)

**Figure 3:**
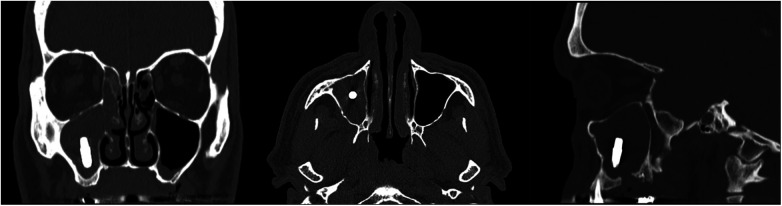
Preoperative computed tomography (CT) scan showing a representative case of a group II ODS. Key anatomical findings include the complete opacification of the maxillary sinus, secondary obstruction of the ostiomeatal complex (OMC), and the presence of a migrated dental implant acting as a septic foreign body within the sinus cavity.

It was essential in management to be able to differentiate between a functional implant being part of a sinus reaction and an implant that had become irreversibly septic foreign body. The literature attests that the simple encroachment of an implant apex in a sinus cavity (“procidence”) is no pathology in itself. As shown by Ragucci et al. (13), once the implant is below the floor of the sinus cavity, osseointegration and long-term survival can be preserved so far as Schneiderian membrane is healed or epithelialized over its tip but without active inflammation. In those cases of asymptomatic, “incidental” protrusion, there is no reason to treat the implant as a foreign body (Figure 3).

However, once an infection develops, the landscape changes significantly. We followed specific clinical and radiological criteria to select non-vital foreign body implants that had to be removed:

- **Mobility (Loss Of Integration):** Complete loss of osseointegration and an infected fibrous capsule can be observed clinically as mobility. In these cases the implant acts as a movable foreign body and a conduit for injecting bacteria into the sinus cavity during mastication (14).
- **Radiographic Osteolysis:** Peri-implant radiolucency (halo effect) on preoperative CT scanning is pathognomonic of apical peri-implantitis or severe marginal bone loss. As suggested by Chen et al. (6), this radiographic sign demonstrates biofilm colonization of the implant surface. Once the rough, contaminated Ti surface has been established it is nearly impossible to sterilize *in situ* and thus serves as a “bacterial reservoir” (sequestrum) that contributes to chronic sinusitis even with maximal medical or endoscopic therapy (15–17).

In our cohort, the high rate of implant removal (Group II) reflects strict adherence to these criteria: implants were only preserved if they were stable, radiologically integrated without peri-implant osteolysis, and the sinus pathology could be managed solely by restoring ventilation via FESS. On the contrary, all affected mobile implants or presenting marked radiolucency were considered active foreign bodies and extracted to obtain resolution of sinonasal infection.

A crucial finding in our series was the high prevalence of osteomeatal complex (OMC) obstruction (68.9%) associated with unilateral opacification. This confirms that even when the primary “noxa” is located at the floor of the sinus (e.g., an implant apex), the resulting inflammation often triggers a blockade of the natural ostium (6). This creates a closed, hypoxic environment that fosters anaerobic bacterial overgrowth, likely explaining why medical therapy failed in 100% of our cases. The restoration of physiological ventilation is therefore a key step in the treatment protocol (3, 6).

### 4.3 Surgical management strategy

The management of odontogenic sinusitis (ODS) related to implantology presents unique challenges compared to classic dental etiologies. The high incidence of oroantral communications (OAC) in our total sample (60%) and the presence of foreign bodies in 62.2% of patients strongly influenced our surgical strategy. The results of our study highlight a clear trend toward the combined simultaneous approach, which was the most frequently adopted strategy (57.8% of the total cohort). However, this trend must be interpreted within the context of our selection bias, as our cohort strictly represents patients who had already failed conservative medical management and required surgical intervention. This finding strongly aligns with the protocol proposed by Felisati et al. (1) and corroborated by our group (3), which posits that addressing the sinus pathology and the odontogenic source in a single surgical session represents a potentially effective strategy for complex cases. The rationale lies in the concurrent presence of OMC obstruction—requiring FESS to restore ventilation—and an OAC or a displaced foreign body requiring intraoral access for closure or retrieval. In this context, addressing only one side of the problem is insufficient: the Endoscopic (FESS) component is essential to remove widely dispersed debris that an oral approach cannot reach, while the Oral approach is mandatory to manage the dental source and seal the oral cavity.

**Group II:** Regarding Group II, our data allow for a critical discussion on the fate of the dental implant. The nasal-only approach (FESS) was utilized in 13.5% of this subgroup (according to our sub-analysis), supporting the paradigm introduced by Chen et al. (6), which suggests that FESS alone can be curative in selected cases where the implant remains stable and no OAC is present. We adopted specific criteria for removal:

1. **Loss of Integration (Mobility):** Implants exhibiting mobility were considered active foreign bodies surrounded by fibrous/infected tissue, pumping bacteria into the sinus.
2. **Radiological Osteolysis:** The presence of peri-implant radiolucency (halo effect) was interpreted as a sign of biofilm colonization, rendering the surface impossible to sterilize *in situ*.

Consequently, the high rate of approaches involving oral surgery in Group II (Combined 59.5% + Oral-only 27.0% = 86.5%) reflects the fact that, in our experience, referred patients typically present with implants that are too compromised to be preserved. Attempting to preserve a septic implant in the presence of chronic sinusitis carries a high risk of recurrence (5).

**Group I:** Specific consideration must be given to complications arising from sinus floor elevation (Group I), observed in 17.8% of patients. In this subgroup, the combined approach was utilized in 50% of cases. This strategy is strongly influenced by the physicochemical nature of the graft material involved in the infection (7, 8):

- Fluid/Gel formulations, often used in crestal approaches, may theoretically allow for natural clearance via the ostium if migration occurs, potentially causing transient acute reactions rather than permanent blockage.
- Particulate Grafts (Granules) pose greater risks. Micro-granules, due to their small mass, may theoretically be cleared more easily. Macro-granules, conversely, tend to aggregate. If they migrate towards the osteomeatal complex, they can create a “dam effect”, mechanically interlocking at the ostium and causing an immediate, total blockade of sinus ventilation (9, 10). Furthermore, the lack of osseointegration transforms the graft into a porous foreign body. Unlike autogenous bone which may resorb, synthetic or xenogeneic particles (especially non-resorbable ones) persist indefinitely, acting as a scaffold for bacterial biofilm. This explains why an oral approach alone is often insufficient in Group I: it allows for the removal of the bulk of the graft but fails to reach granules migrated into the ostium or ethmoidal recesses, making FESS indispensable for complete resolution.
- Our surgical decision-making was strictly guided by anatomical and pathological findings. The exclusive oral approach was utilized only when pre-operative imaging confirmed a perfectly patent osteomeatal complex (OMC). On the other hand, the exclusive nasal approach (FESS alone) was employed in a specific subset of patients characterized by the complete absence of foreign bodies, oroantral communications, or Schneiderian membrane perforations. In these cases, the pathogenesis was not driven by a septic graft, but rather by a residual inflammatory reaction following membrane elevation that failed to heal due to an inefficiently functioning maxillary sinus and impaired drainage.

**Figure 4:**
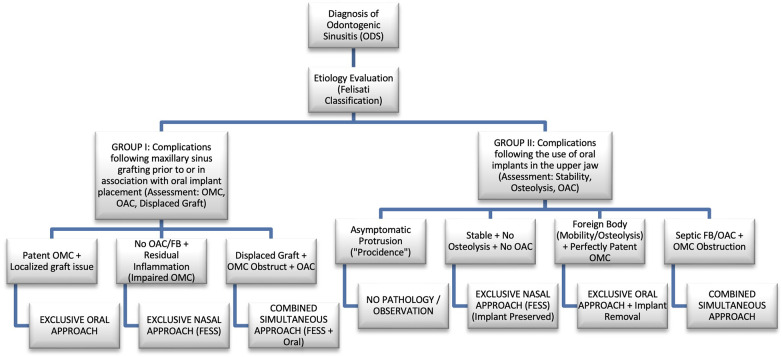
Summary of the institutional surgical decision-making process applied in the present cohort. This algorithm reflects the authors’ retrospective institutional experience; it has not undergone external validation and can be tailored based on specific clinical conditions, available technology, and surgical expertise.

To enhance the clinical utility of these findings, we have summarized our institutional surgical decision-making process in a flowchart (Figure 4) with its corresponding operational selection parameters structured in Table 3. This algorithm is based on our center's internal guidelines and retrospective clinical experience. It should be interpreted as a descriptive framework rather than a rigid, universal treatment pathway. In real-world practice, this approach can be highly tailored based on the patient's specific clinical conditions, the availability of dedicated instrumentation and technologies, and the multidisciplinary skills and experience of the surgical team.

**Table 3:** Operational clinical and radiological criteria for surgical treatment selection.

Surgical approach	Key operational criteria and indications
Exclusive Endoscopic Approach (FESS)	OMC obstruction AND absence of an oroantral communication (OAC) AND stable dental implants (no peri-implant osteolysis) where the infection is confined to the sinus cavity.
Exclusive Oral Approach	Patent OMC (clear on CT and endoscopy) AND presence of an OAC requiring flap closure OR presence of Foreign body OR localized alveolar bone/graft infection without sinonasal tract extension.
Combined Simultaneous Approach	Co-existence of OMC obstruction (requiring FESS) AND an active OAC or foreign body (implant/graft material) requiring trans-oral removal and ridge debridement.
Implant Removal Criteria	Clinically mobile dental implant OR severe peri-implant radiographic radiolucency OR completely dislocated implant
Implant Preservation Criteria	Clinically stable implant without peri-implant radiolucency.

### 4.4 Limitations

This study presents several limitations that must be acknowledged. Primarily, the retrospective and single-center design limits the generalizability of the findings. As our institutional database specifically captures surgically managed cases, the exact number of patients who were evaluated for ODS but successfully treated with medical therapy alone was not available for this analysis. Furthermore, there is an inherent selection bias: because our cohort was derived exclusively from a surgical database, it represents the more severe, refractory end of the ODS spectrum, as patients who successfully responded to initial medical therapy were not captured. Finally, the relatively small sample size—particularly in Group I—and the lack of long-term radiological follow-up beyond the 3-month clinical and endoscopic assessment highlight the need for larger, prospective, multi-center trials to validate these surgical algorithms. Furthermore, our short-term evaluation at 3 months demonstrated a high primary success rate (97.8%), with only one recurrence (a reopened OAC in Group II) that was successfully managed with a secondary procedure. While this reflects excellent short-term outcomes following the removal of the primary odontogenic noxa, this retrospective evaluation likely underestimates minor, transient post-operative morbidities and cannot account for potential long-term recurrences beyond the follow-up period. Our study is also limited by the lack of systematic intraoperative microbiological findings. Although the polymicrobial and anaerobic nature of odontogenic sinusitis is well-documented in literature, cultures were not routinely collected in this retrospective cohort. Future prospective trials should mandate routine microbiological sampling to better support targeted antimicrobial stewardship. Moreover, potentially relevant patient-level variables influencing peri-implant health and wound healing such as smoking status, diabetes, or other systemic diseases were not systematically analyzed. Moreover, regarding cohort selection, our study specifically focuses on patients who were refractory to previous medical therapy and required surgical intervention. While these data are not intended to estimate the true prevalence of various ODS phenotypes within the general population of dental implant patients, this cohort accurately reflects the real-world demographics of complex, severe cases referred to a specialized center. These are precisely the challenging scenarios where an advanced, tailored surgical algorithm is most needed. Furthermore, regarding objective scoring systems, our retrospective protocol did not systematically employ rhinogenic-specific metrics such as the Sino-Nasal Outcome Test (SNOT-22), Lund-Mackay CT staging, or Lund-Kennedy endoscopic scores. While these standardized tools are highly valuable for tracking classical primary rhinosinusitis, we deemed them less clinically indicative for a secondary, iatrogenic entity like implant-related ODS, which is inherently characterized by a maxillary pathology. Instead, our diagnostic and postoperative assessments relied strictly on detailed multiplanar CT findings and direct middle meatus nasal endoscopy. Finally, the relatively short follow-up period of three months represents another limitation. While this timeframe is generally considered adequate to evaluate primary sinonasal mucosal healing, successful OAC closure, and the resolution of acute symptoms, it is insufficient to assess the long-term survival of the preserved dental implants or the incidence of late recurrences. Future studies with extended follow-up are warranted

## 5 Conclusion

Our retrospective analysis suggests that there is no “one-size-fits-all” treatment for implant-related ODS; rather, achieving complete clinical resolution demands a tailored surgical management guided by the patient's specific phenotypic profile. Based on our clinical experience, a combined simultaneous approach was frequently utilized to manage complex cases presenting with OMC obstruction and foreign bodies. Although our retrospective design precludes establishing the absolute superiority of one strategy over another, our experience-based findings highlight the combined approach as a highly reliable and effective option for complex cases. Exclusive surgical approaches (nasal or oral) were also highly effective when properly tailored to carefully selected patients. Consistent with the paradigm described by Chen et al. (6), FESS alone represents a valid option for implant preservation in selected cases without OAC. However, the overarching complexity of pre-implant and implant complications often requires a more aggressive, multidisciplinary surgical intervention to safely execute this tailored strategy. Further prospective, multi-center studies are needed to establish robust, evidence-based treatment paradigms for these complex iatrogenic complications.

## Data Availability

The raw data supporting the conclusions of this article will be made available by the authors, without undue reservation.
